# Growth factors in synaptic function

**DOI:** 10.3389/fnsyn.2013.00006

**Published:** 2013-09-18

**Authors:** Vivian Y. Poon, Sojoong Choi, Mikyoung Park

**Affiliations:** ^1^Neuroscience and Behavioral Disorders Program, Duke-NUS Graduate Medical SchoolSingapore, Singapore; ^2^WCI, Center for Functional Connectomics, Brain Science Institute, Korea Institute of Science and TechnologySeoul, South Korea; ^3^Department of Neuroscience, University of Science and TechnologyDaejeon, South Korea

**Keywords:** netrin, Wnt, TGF-β, TNF-α, synaptogenesis, synaptic transmission and plasticity

## Abstract

Synapses are increasingly recognized as key structures that malfunction in disorders like schizophrenia, mental retardation, and neurodegenerative diseases. The importance and complexity of the synapse has fuelled research into the molecular mechanisms underlying synaptogenesis, synaptic transmission, and plasticity. In this regard, neurotrophic factors such as netrin, Wnt, transforming growth factor-β (TGF-β), tumor necrosis factor-α (TNF-α), and others have gained prominence for their ability to regulate synaptic function. Several of these factors were first implicated in neuroprotection, neuronal growth, and axon guidance. However, their roles in synaptic development and function have become increasingly clear, and the downstream signaling pathways employed by these factors have begun to be elucidated. In this review, we will address the role of these factors and their downstream effectors in synaptic function *in vivo* and in cultured neurons.

## Introduction

Human perception, learning, and memory are only possible when the nervous system is functioning normally. The primary building blocks of the nervous system are neurons–specialized cells that form connections, or synapses, with specific targets. Loss or malfunction of synapses leads to mental retardation, schizophrenia, and neurodegenerative diseases like Alzheimer's or Parkinson's disease.

As a functional synapse is a fundamental requirement for the brain to process any task, synaptic function is tightly regulated. This regulation occurs at multiple steps, such as recruitment and assembly of molecular machinery, synapse formation and stabilization, coordinated release of neurotransmitters, downstream signaling of receptors, maintenance, plasticity, and eventual loss of the synapse.

To study synaptic function, neurobiologists have utilized multiple model systems, including *C. elegans*, *Drosophila*, the vertebrate neuromuscular junction (NMJ), primary mammalian neurons, brain slice cultures, and rodent models. Pioneering work in invertebrates led to the identification of novel roles for growth factors in synaptic function (Zhang et al., 1997; Aberle et al., 2002; Chin et al., 2002; Marques et al., 2002; Packard et al., 2002; McCabe et al., 2003; Ziel and Sherwood, 2010), and subsequent studies have demonstrated similar synaptic functions for these growth factors in mammals (Krieglstein et al., 2011; Salinas, 2012; Horn et al., 2013).

Through these studies, the role of growth factors such as netrin, Wnt, transforming growth factor-β (TGF-β), and tumor necrosis factor-α (TNF-α) in synaptogenesis, synaptic transmission, and plasticity is gradually being elucidated. Netrin, Wnt, and TGF-β both enhance and suppress synaptogenesis, and their effects are mediated through a variety of pathways (Shen and Cowan, 2010; Krieglstein et al., 2011; Koles and Budnik, 2012; Salinas, 2012). In addition, the netrin receptor Deleted in Colorectal Cancer (DCC) is implicated in synaptic plasticity (Horn et al., 2013) while members of the Wnt superfamily enhance synaptic function *in vivo* and *in vitro* primarily through the planar cell polarity (PCP) and calcium Wnt signaling pathways (Koles and Budnik, 2012; Salinas, 2012). In contrast, the TGF-β superfamily and TNF-α enhance excitatory synaptic transmission, while suppressing inhibitory synaptic transmission (Krieglstein et al., 2011; Santello and Volterra, 2012). In this review, we will focus on the function of netrin, Wnt, TGF-β, and TNF-α in the various aspects of synaptic function and the downstream signaling pathways employed. Roles of other growth factors like brain-derived neurotrophic factor (BDNF), fibroblast growth factor (FGF), and glial cell line-derived neurotrophic factor (GDNF) are discussed elsewhere (Shen and Scheiffele, 2010; Wu et al., 2010; Duarte et al., 2012; Park and Poo, 2013).

## Netrin

The netrin family of laminin-related proteins is known for its critical role in axon guidance during neuronal development. Over the past two decades, netrins have been implicated in diverse processes in multiple tissues, including cell adhesion (Baker et al., 2006), cell survival (Ko et al., 2012), and tumorigenesis (Arakawa, 2004). Within the nervous system, there is emerging evidence for netrins as novel regulators of synaptogenesis and synaptic function (Shen and Cowan, 2010; Flores, 2011). As it is challenging to isolate a synaptogenic function of netrin that is independent of its function in guidance, the role for netrin at synapses has mostly been addressed in simple and genetically tractable systems like *C. elegans*, *Drosophila*, and *Xenopus* (Winberg et al., 1998; Colon-Ramos et al., 2007; Poon et al., 2008; Manitt et al., 2009). Nonetheless, as tools that allow temporal-specific perturbation of netrins or their signaling components become available (Lai Wing Sun et al., 2011; Horn et al., 2013), more studies addressing the synaptogenic role of netrin should follow.

The founding member of the netrin family, uncoordinated-6 (UNC-6), was first identified as a component of the extracellular matrix that guides dorsoventral migration in *C. elegans* (Ishii et al., 1992). In mammals, the netrin family is composed of five members: netrin 1, 3 and 4, which are secreted and highly conserved, and netrin G1 and G2, which are glycophosphatidylinositol (GPI)-linked and vertebrate-specific. Netrin signaling is transduced through receptors such as DCC/Frazzled/UNC-40, neogenin, the UNC-5 family, and Down syndrome cell adhesion molecule (DSCAM) (Lai Wing Sun et al., 2011). The effectors that lie downstream of DCC, neogenin, and UNC-5 receptors comprise regulators of the cytoskeleton like the Rho family of GTPases, Src-family kinases, focal adhesion kinase and microtubule-associated proteins (Li et al., 2004b; Rajasekharan and Kennedy, 2009). In contrast, netrin Gs bind to netrin G ligands (NGLs) NGL-1/LRRC4C and NGL-2/LRRC4 (Nakashiba et al., 2000, 2002; Lin et al., 2003; Kim et al., 2006). The NGL family also includes NGL-3, a member that does not bind netrin Gs. As these membrane-anchored netrins and their ligands are less characterized, their signaling pathways remain unclear.

Though netrins and their receptors are widely studied for their role in nervous system development, they are continually expressed throughout adulthood (Livesey and Hunt, 1997; Manitt and Kennedy, 2002; Horn et al., 2013), suggesting that they play additional roles that are distinct from early developmental events. In addition, both netrin 1 and its receptor DCC are present in synaptosomes (Horn et al., 2013) and may thus act locally at synapses. Netrin Gs are similarly highly expressed in the adult brain and exhibit complex non-overlapping expression patterns (Nakashiba et al., 2002; Yin et al., 2002).

Netrin signaling in the nervous system is further altered when neuronal activity is perturbed. Levels of netrin receptors and netrin G2 are regulated by psychostimulant drugs (Yetnikoff et al., 2007; Argento et al., 2012), endocannabinoid receptor antagonists (Argaw et al., 2011), and epilepsy-induced activity (Pan et al., 2010). Amphetamine treatment elevates the expression of DCC and UNC-5 receptors in the mesocorticolimbic dopamine system in adult rats (Yetnikoff et al., 2007), while methylphenidate lowers the expression of DCC in the ventral tegmental area (VTA) of adult mice (Argento et al., 2012). It is intriguing to note that this down-regulation of DCC levels is associated with diminished sensitivity to cocaine (Argento et al., 2012). Taken together, these studies suggest that drugs that induce plasticity in the dopamine system regulate netrin receptor levels. Treatment of cultured primary cortical neurons with endocannabinoid receptor antagonists elevates surface expression of DCC (Argaw et al., 2011), suggesting that synaptic transmission of endocannabinoids regulates DCC activity. Netrin G2 levels are also elevated in the cortex of epileptic patients and mice (Pan et al., 2010), indicating that netrin G2 expression may be regulated by alterations in neuronal activity induced by epilepsy. While the significance, consequence, and underlying mechanisms of the regulation of netrin and its receptors are still being addressed, these studies provide preliminary evidence for a putative role for netrins in synaptic function. We will next explore the known functions of netrins in synaptogenesis, synaptic transmission, and plasticity.

### Role of netrins in synaptogenesis

Work in *Drosophila* motor neurons was the first to suggest a synaptogenic role for netrins. Overexpressing netrin in ventral muscles leads to DCC/frazzled-dependent formation of ectopic synapses in the transverse nerve in flies (Winberg et al., 1998). Similarly, addition of netrin into the *Xenopus* optic tectum augments the number of pre-synaptic sites in retinal ganglion cell axons in a DCC-dependent manner (Manitt et al., 2009). However, the downstream signaling components remain unknown.

**Figure 1:**
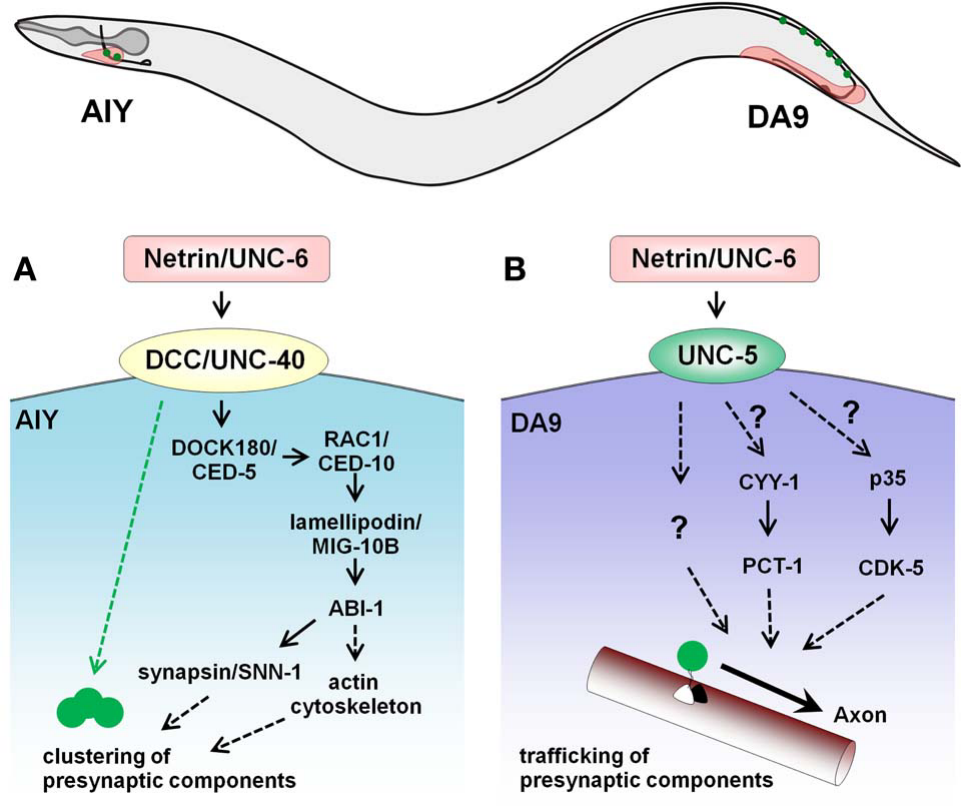
**Opposing roles of netrin/UNC -6 on synaptogenesis in the *C. elegans* AIY interneuron and DA9 motor neuron. (A)** In the head region, glial sheath cells secrete UNC-6 and promote pre-synaptic assembly in AIY. UNC-6 signals through the DCC/UNC-40 receptor in AIY and recruits DOCK180/CED5 to pre-synaptic sites, leading to actin cytoskeleton remodeling and the pre-synaptic vesicle clustering. In addition, activation of UNC-40 clusters active zone proteins in a DOCK180/CED5-independent fashion (green dotted arrow). **(B)** In the tail, UNC-6 secreted by the ventral muscles acts through the UNC-5 receptor to prevent mislocalization of pre-synaptic components to the dendrite of the DA9 motor neuron. Pctaire kinase PCT-1, cyclin CYY-1, cyclin-dependent kinase CDK-5 and its activator p35 also regulate the kinesin/dynein-mediated localization of pre-synaptic components and may act downstream of UNC-6. The black arrow indicates the trafficking of components to the axon.

Subsequent studies in *C. elegans* provided further evidence for the synaptogenic function of netrin (Figure 1A). Secretion of netrin/UNC-6 by glia coordinates innervation between AIY and RIA, two interneurons that mediate thermotaxis (Colon-Ramos et al., 2007). Loss of netrin/UNC-6 or its receptor DCC/UNC-40 leads to defects in pre-synaptic assembly in AIY without affecting axon guidance. The DCC/UNC-40 receptor interacts with and localizes DOCK180/CED-5, which signals through Rac1/CED-10, lamellipodin/MIG-10B, and a component of Wiskott-Aldrich syndrome protein family, Abelson-interacting protein-1 (ABI-1) to regulate the actin cytoskeleton at pre-synaptic sites (Stavoe and Colon-Ramos, 2012; Stavoe et al., 2012). In addition, synaptic vesicle clustering is regulated through synapsin/SNN-1, which lies downstream of ABI-1 and lamellipodin/MIG-10B. These studies in the *C. elegans* AIY interneuron have thus elucidated the signaling effectors responsible for netrin-mediated synaptogenesis.

In addition to promoting synaptogenesis, netrin/UNC-6 expressed by ventral tissue also inhibits ectopic synapse formation in *C. elegans* (Figure 1B). Removal of netrin/UNC-6 or its receptor UNC-5 results in mislocalization of pre-synaptic components to the ventral dendrite of the DA9 motor neuron, and this effect is independent of guidance defects (Poon et al., 2008). Considering how netrin/UNC-6 drives synaptogenesis in AIY through the DCC/UNC-40 receptor, it is not surprising that netrin/UNC-6 functions in an opposite manner in DA9 when signaling through UNC-5. Since several intracellular regulators like the novel cyclin CYY-1, a cyclin-dependent kinase CDK-5, and the Pctaire kinase PCT-1 govern proper localization of pre-synaptic components in DA9 (Ou et al., 2010), it is possible that these factors lie downstream of netrin/UNC-6-UNC-5 signaling.

Apart from regulating pre-synapse formation, the netrin receptor DCC/UNC-40 also directs differentiation of muscles that are post-synaptic to motor axons and egg-laying motor neurons in *C. elegans*. Absence of this receptor leads to a reduction in post-synaptic muscle arm extensions (Alexander et al., 2009) and abolishes vulval muscle arms (Li et al., 2013a). In both instances, however, netrin/UNC-6 is not required. Taken together, the above studies demonstrate that netrin and its receptors modulate synaptogenesis in invertebrates and *Xenopus*. However, this begs the question: does netrin function similarly in mammals?

The role of netrin 1 and its receptor DCC in the mesocorticolimbic dopamine system has been explored (Flores, 2011). Adult mice lacking DCC have reduced dendritic spine densities in layer V pyramidal neurons in the medial prefrontal cortex (Grant et al., 2007; Manitt et al., 2011), suggesting that DCC is required for post-synaptic differentiation. These mice also exhibit defects in pubertal maturation of synaptic connectivity of dopaminergic neurons in this brain area, where numbers of tyrosine hydroxylase-positive varicosities are elevated (Manitt et al., 2011). Further work is needed to confirm if these varicosities are functional pre-synaptic terminals and if this is a secondary effect of axon misguidance. In addition, knocking down DCC in dopaminergic neurons *in vitro* suppresses the formation of autaptic axon terminals (Xu et al., 2010), consistent with a pro-synaptogenic role for DCC. While studies in *C. elegans* have provided some insights into netrin-mediated signaling pathways involved in regulating synaptogenesis, further studies in the mammalian central nervous system (CNS) are pertinent to elucidating the synaptogenic function of netrins.

Unlike secreted netrins, which act as both positive and negative regulators of synaptogenesis, the netrin G2 receptor NGL-2 primarily promotes synaptogenesis in cultured hippocampal neurons (Kim et al., 2006). NGL-2 was first identified as a novel binding partner of the post-synaptic scaffolding protein PSD-95. Overexpressing NGL-2 elevates the number of dendritic spines while knocking it down causes a loss in excitatory, but not inhibitory synapses. In hippocampal slices, removing NGL-2 leads to selective loss of spines in CA1 dendrites in the stratum radiatum, and spine formation requires NGL-2-netrin G2 binding (Denardo et al., 2012). Intriguingly, netrin G2 knockout mice have no detectable anomalies in the density of PSD-95 clusters in the hippocampus (Nishimura-Akiyoshi et al., 2007). Work in cultured hippocampal neurons further indicates that NGL-2-induced post-synaptic differentiation occurs via multiple mechanisms that are PSD-95-dependent or -independent (Kim et al., 2006). In addition to driving post-synaptic differentiation, NGL-2, like the post-synaptic cell adhesion molecule neuroligin, is sufficient to induce pre-synaptic differentiation (Kim et al., 2006). NGL-2 likely binds to netrin G2 and other factors to mediate this process since netrin G2 alone is insufficient to induce post-synaptic differentiation. Understanding the signaling pathways downstream of NGL-2 will be critical for comprehending the mechanisms of NGL-2 function.

### Role of netrins in synaptic transmission and plasticity

Given that netrins and their signaling components are expressed in adulthood (Livesey and Hunt, 1997; Manitt and Kennedy, 2002; Horn et al., 2013) and regulate synaptogenesis (Winberg et al., 1998; Kim et al., 2006; Colon-Ramos et al., 2007; Poon et al., 2008; Manitt et al., 2009; Flores, 2011), one may expect netrins to regulate synaptic transmission and plasticity. Several groups employing DCC-deficient mice and mammalian hippocampal cultures have attempted to explore this possibility.

Mice lacking DCC have altered dopamine transmission and are insensitive to the stimulant drug of abuse amphetamine (Grant et al., 2007; Yetnikoff et al., 2007, 2010). These mice exhibit enhanced amphetamine-induced dopamine release in the medial prefrontal cortex, but display the opposite response in the nucleus accumbens (Grant et al., 2007). A reduction in DCC also suppresses the rewarding effects of amphetamine on behavior and neuronal activity (Grant et al., 2007), and this effect is likely due to loss of DCC activation in the VTA (Yetnikoff et al., 2010). A deficiency in DCC also abolishes the amphetamine-induced increase in the expression of dendritic spine-associated protein spinophilin in the VTA (Yetnikoff et al., 2010).

A recent study further implicates DCC in synaptic plasticity in forebrain pyramidal neurons in the adult (Horn et al., 2013). Forebrain neurons in which DCC is deleted late in development had shorter dendritic spines, impaired long-term potentiation (LTP) but not long-term depression (LTD), and diminished expression of N-methyl-D-aspartate-type glutamate receptor (NMDAR) subunit GluN2B, Src, phosphorylated phospholipase C γ1, and phosphorylated Src family kinase Fyn. As deficits in LTP displayed by the DCC knockout mouse are rescued by Src activation or NMDAR function enhancement, it is likely that DCC regulates NMDAR-dependent plasticity through Src (Horn et al., 2013).

Using heterozygous mutants or conditional knockout mice, the previous studies showed that DCC is required for plasticity in the limbic system and the hippocampus. What about the ligand? To examine if netrin 1 affects synaptic function and plasticity, Bayat and colleagues infused netrin 1 into the hippocampus of mice after cerebral ischemia (Bayat et al., 2012). This treatment improved spatial memory impairment, basal evoked potential, and LTP, suggesting that netrin 1 is sufficient to enhance synaptic transmission. However, the underlying mechanism was not determined and the effects observed may be secondary to a pro-survival function of netrin 1. Nonetheless, this is the first study investigating an *in vivo* role for netrin in mammalian synaptic function and plasticity.

Apart from secreted netrins and their receptors, NGL-2 is also required for proper synaptic transmission. As previously described, NGL-2 drives synaptogenesis in cultured hippocampal neurons. Knocking down NGL-2 diminishes the frequency, but not the amplitude of miniature excitatory post-synaptic currents (mEPSCs) and has no effect on inhibitory currents (Kim et al., 2006). In hippocampal slices, removal of NGL-2 reduces synaptic transmission at Schaffer collateral synapses in the stratum radiatum of the CA1 region (Denardo et al., 2012). Hence, in addition to promoting synaptogenesis, NGL-2 drives synaptic transmission in distinct regions in the hippocampus, and regulates excitatory but not inhibitory synaptic function.

## Wnts

First identified as key regulators of embryonic development, Wnt proteins have gained prominence over the past decade for their role in synapse formation and function in both the central and peripheral nervous system (Budnik and Salinas, 2011; Koles and Budnik, 2012; Salinas, 2012). These secreted lipo-glycoproteins are evolutionarily conserved and the mammalian genome comprises 19 Wnt genes (Willert and Nusse, 2012).

To achieve a wide spectrum of functions, Wnt proteins act through a diverse number of pathways—the canonical, divergent canonical, PCP, calcium Wnt signaling, and Frizzled nuclear import (FNI) pathways (Kuhl et al., 2000; Mlodzik, 2002; Ciani et al., 2004; Logan and Nusse, 2004; Speese and Budnik, 2007). These pathways lie downstream of the seven-pass transmembrane Frizzled receptors, and with the exception of the FNI pathway, activate the scaffolding protein Dishevelled (Dvl). In the canonical pathway, Dvl inhibits the Axin/Adenomatous Polyposis Coli (APC)/Glycogen synthase kinase 3β (Gsk3β) complex, and β-catenin is imported into the nucleus where it activates gene transcription. In the divergent pathway, inhibition of Gsk3β leads to decreased phosphorylation and augmented activity of microtubule-associated proteins. In the PCP pathway, Dvl regulates the cytoskeleton by activating the small Rho GTPases RhoA and Rac1, and c-Jun-amino-terminal kinase (JNK). In the calcium Wnt signaling pathway, Dvl increases intracellular calcium levels, thus activating multiple targets, including calcium/calmodulin-dependent protein kinase II (CaMKII), protein kinase C (PKC), and calcineurin, which results in the nuclear import of nuclear factor of activated T-cells (NFAT). In the FNI pathway, Frizzled-2 is internalized, processed, and imported into the nucleus. Though less characterized, Wnts also signal through members of the receptor tyrosine kinase-like orphan receptor (ROR) and the tyrosine kinase-like receptor Derailed (Drl)/Ryk families. Two recent reviews describe the Wnt signaling pathways in further detail (Koles and Budnik, 2012; Mulligan and Cheyette, 2012).

Given their importance in neuronal development and function, it is not surprising to note that Wnt ligands and their signaling components are present in neurons and regulated by activity. Neuronal activity-mediated regulation of Wnt signaling is prevalent in systems ranging from the *C. elegans* and *Drosophila* NMJ to the vertebrate CNS. Neuronal stimulation leads to secretion of the *C. elegans* Wnt ligand CWN-2 (Jensen et al., 2012), as well as release of the *Drosophila* Wnt1 ligand Wingless (Wg) from synaptic boutons in the larval NMJ (Ataman et al., 2008) and the fly olfactory sensory neuron (Chiang et al., 2009). In a central serotonergic neuron in *Drosophila*, activity triggers Wnt signaling and leads to dendritic refinement (Singh et al., 2010). Similarly, during activity-dependent dendrite development in hippocampal neurons, activity elevates Wnt release (Yu and Malenka, 2003) and Wnt2 transcription (Wayman et al., 2006). Wnt3a is also released at synapses in the hippocampus during tetanic stimulation (Chen et al., 2006). Altering activity with exposure to different environments or learning paradigms also changes Wnt levels in the hippocampus. Wnt7a/b levels in post-synaptic CA3 neurons rise when mice are kept in an enriched environment (Gogolla et al., 2009); mice undergoing spatial learning in the Morris water maze have augmented levels of Wnt7, Wnt5, but not Wnt3. Lastly, levels of surface Frizzled-5, a receptor of Wnt7a in the hippocampus, increase with high frequency stimulation (HFS) in a Wnt-dependent fashion (Sahores et al., 2010). Taken together, the tight interplay between neuronal activity and Wnt signaling suggests a critical role for Wnts and their downstream effectors to modulate synaptogenesis, synaptic transmission, and plasticity.

### Role of wnts in synaptogenesis

**Figure 2:**
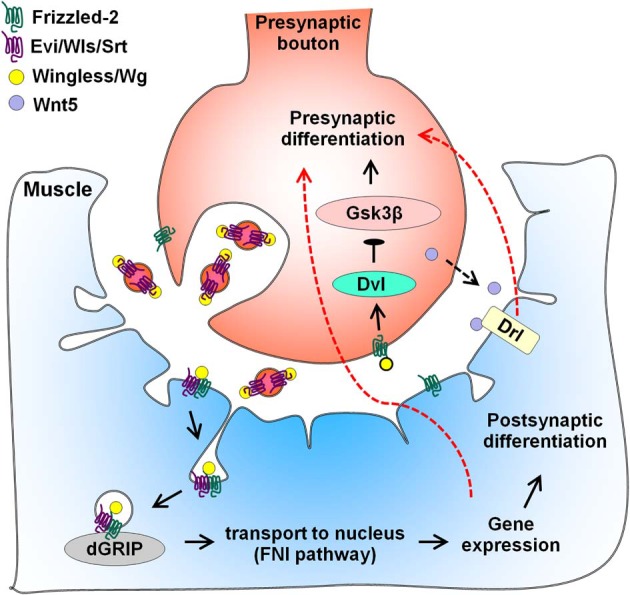
**Wnt regulation of larval NMJ differentiation.** Vesicular release of the Wnt-binding protein Evenness Interrupted/Wntless/Sprinter (Evi/Wls/Srt) facilitates pre-synaptic secretion of Wingless/Wg. In the pre-synaptic bouton, binding of Wg to the Frizzled-2 receptor (DFz2) activates components of the canonical pathway and leads to pre-synaptic differentiation. In the post-synaptic muscle, Wg binds to DFz2, inducing endocytosis of the receptor. As part of the Frizzled nuclear import (FNI) pathway, Evi/Wls/Srt recruits the Wg receptor-interacting protein dGRIP, leading to transport of DFz2 to the nucleus. Entry of DFz2 into the nucleus alters gene expression, promoting post-synaptic, and possibly, pre-synaptic differentiation (red dotted arrow). Additional regulators of Wg signaling excluded from this figure include laminin A, integrin, the HSPG perlecan/trol and HSPG sulfation. In addition, Wnt5 is also secreted by the pre-synaptic bouton (black dotted arrow) and acts through the tyrosine kinase-like receptor Derailed (Drl) to promote pre-synaptic differentiation retrogradely (red dotted arrow).

In the larval NMJ of *Drosophila*, members of the Wnt family promote formation of both pre- and post-synapses (Figure 2). Loss of Wg leads to defective pre- and post-synaptic specializations (Packard et al., 2002). During development, pre-synaptic vesicular release of the Wg-binding protein Evenness Interrupted/Wntless/Sprinter (Evi/Wls/Srt) leads to proper Wg secretion and recruitment of a *Drosophila* glutamate receptor interacting protein (dGRIP) to post-synaptic sites (Korkut et al., 2009). Wg binds the *Drosophila* Frizzled-2 (DFz2) receptor that is located both in the motor neuron and muscle. Several studies indicate that divergent signaling pathways are employed both in the pre-synaptic motor neuron and in the post-synaptic muscle. In the case of the latter, DFz2 is endocytosed from the post-synaptic membrane and transported to the nucleus by binding dGRIP, and this process is required for assembly of the post-synapse (Mathew et al., 2005; Ataman et al., 2006, 2008; Speese et al., 2012). On the pre-synaptic side, Wg signaling involves components of the canonical pathway like Arrow/Low-density lipoprotein receptor-related protein Dvl and Shaggy/Gsk3β to regulate bouton number (Ataman et al., 2008; Miech et al., 2008). Anterograde Wg signaling also modulates NMJ growth through the retrograde signal laminin A and the pre-synaptic integrin pathway (Tsai et al., 2012). Thus, Wg signals bi-directionally and utilizes distinct pathways in pre- and post-synaptic compartments. Recently, Kamimura and colleagues found that bi-directional signaling by Wg is regulated by a secreted heparan sulfate proteoglycan (HSPG) perlecan/trol (Kamimura et al., 2013). Coincidentally, Wg levels are also altered by HSPG sulfation (Dani et al., 2012). In addition to Wg, loss of Wnt5 leads to a reduction in the number of pre-synaptic boutons and suppresses active zone formation (Liebl et al., 2008). Wnt5 signals through its post-synaptic receptor Drl but some of its functions are Drl-independent. Taken together, these studies suggest that Wg and Wnt5 drive synaptogenesis in the fly NMJ.

In the vertebrate NMJ, Wnt3 and Wnt11r enhance synaptogenesis. Wnt3 augments acetylcholine receptor (AChR) clustering in the chick wing NMJ and in cultured myotubes via the non-canonical PCP pathway involving Rac1 activation and Rho signaling (Rattner et al., 1997; Weston et al., 2003; Niehrs, 2006; Henriquez et al., 2008). Similarly, the non-conventional Wnt11r is required for AChR clustering in zebrafish, but acts through the muscle-specific kinase (MuSK)/unplugged receptor and Dvl1 (Jing et al., 2009). In addition to Wnt3 and Wnt11r, Wnt signaling components like Dvl1, Dvl1-interacting protein p21-activated kinase1 (Luo et al., 2002), APC (Wang et al., 2003), and β-catenin (Zhang et al., 2007; Li et al., 2008) are implicated as positive regulators of NMJ development.

Studies in the glutamatergic cerebellar glomerular rosette, a multi-synaptic structure formed between mossy fibers and granule cells, provided the first glimpse into the synaptogenic role of Wnts in the vertebrate CNS. Loss of Wnt7a or Dvl1 delays the maturation of glomerular rosettes and leads to defects in the localization of pre-synaptic markers while expression of Wnt7a in granule cells induces clustering of the pre-synaptic protein synapsin I in mossy fiber axons (Hall et al., 2000; Ahmad-Annuar et al., 2006). Wnt7a similarly stimulates clustering of pre-synaptic markers in hippocampal neurons and acts through the Frizzled-5 receptor (Cerpa et al., 2008; Sahores et al., 2010). In addition to regulating the pre-synapse, Wnt7a signaling also promotes dendritic spine growth and PSD-95 clustering through Dvl1 and CaMKII (Ciani et al., 2011). Lastly, mice exposed to an enriched environment have an increased number of synapses in their hippocampus and this effect is dependent on Wnt7a/b (Gogolla et al., 2009).

Apart from Wnt7a/b, Wnt5a also regulates synaptogenesis in the hippocampus. There are conflicting reports on the effect of Wnt5a on the pre-synapse while the role of Wnt5a at the post-synapse is less controversial. Several studies report that Wnt5a increases clustering of pre-synaptic proteins and synaptic contacts (Varela-Nallar et al., 2012), and acts through ROR1/2 receptors to promote pre-synaptic assembly in cultured hippocampal neurons (Paganoni et al., 2010). However, other studies indicate that Wnt5a decreases the number of pre-synaptic terminals or has no effect on hippocampal neurons (Davis et al., 2008; Farias et al., 2009). In dendrites, Wnt5a increases calcium levels, spine size, and spine number during development (Varela-Nallar et al., 2010), and clusters PSD-95 through a JNK-dependent signaling pathway (Farias et al., 2009). On top of regulating synapse formation in the pre- and post-synaptic compartments of excitatory synapses, Wnt5a also augments the insertion and clustering of γ-aminobutyric acid A (GABA_A_) receptors in hippocampal neurons by activating CaMKII (Cuitino et al., 2010).

Like Wnt7 and Wnt5a, Wnt3 also induces pre-synaptic protein clustering in hippocampal neurons and drives synapse formation between sensory and motor neurons in the spinal cord. Through the pre-synaptic Frizzled-1 receptor, Wnt3a elevates the number of Bassoon clusters in axons (Varela-Nallar et al., 2009). In motor neurons, Wnt3 secretion induces synapsin clustering and regulates terminal arborization of sensory neurons in a Gsk3β-dependent manner (Krylova et al., 2002). Hence, Wnts, particularly Wnt7a/b, Wnt5a, and Wnt3, regulate synaptogenesis in vertebrate cerebellar, hippocampal, and spinal neurons through diverse signaling mechanisms. Whereas Wnt7a signals through Frizzled-5, Dvl1, and/or CaMKII, Wnt5a signaling occurs via ROR receptors, JNK, or CaMKII, and Wnt3 acts through Gsk3β.

While the previously described Wnt ligands are generally positive regulators of synaptogenesis, other Wnt ligands in *Drosophila*, *C. elegans*, and mice also negatively regulate synaptogenesis. Wnt4 is preferentially expressed in the *Drosophila* muscle cell M13. Absence of Wnt4, its receptor DFz2, Drl2, or Dvl results in the formation of ectopic synapses by motor neuron 12 onto M13 (Inaki et al., 2007). Similarly, in the *C. elegans* cholinergic motor neuron DA9, LIN-44, a Wnt ligand secreted by the tail hypodermal cells, inhibits ectopic synapse formation in the posterior segment of the neuron through the LIN-17/Frizzled receptor and DSH-1/Dvl (Klassen and Shen, 2007). Since other known canonical, PCP, calcium Wnt signaling pathway effectors have no effect, a pathway comprising novel mediators may be employed. In vertebrates, Wnt3a inhibits post-synapse formation by reducing AChR clustering in cultured myotubes through the canonical pathway involving β-catenin (Wang et al., 2008). Taken together, Wnts both enhance and suppress synaptogenesis through the engagement of both canonical and non-canonical pathways (Table 1).

**Table 1:** **Known functions of Wnts in synaptogenesis and synaptic function**.

**Wnt**	**System**	**Function**	**Pathway**	**References**
Wg/Wnt1	*Drosophila* NMJ	Pre-synaptic differentiation	Canonical (Arrow/LRP, Dvl, Gsk3β)	Packard et al., 2002; Ataman et al., 2008; Miech et al., 2008
		Post-synaptic differentiation	FNI	Mathew et al., 2005; Ataman et al., 2006; Speese et al., 2012
		Activity-dependent synaptic growth	Unclear	Ataman et al., 2008
Wnt4	*Drosophila* NMJ	Inhibit ectopic synapses	Dfz2, Drl2, Dvl	Inaki et al., 2007
Wnt5	*Drosophila* NMJ	Pre-synaptic differentiation	Drl	Liebl et al., 2008
		Pre-synaptic transmission	Not through Drl	Liebl et al., 2008
LIN-44	*C. elegans* DA9 neuron	Inhibit ectopic synapses	LIN-17/Fz, Dvl	Klassen and Shen, 2007
CWN-2	*C. elegans* NMJ	AChR clustering, synaptic transmission	LIN-17/Fz, Dvl, ROR/CAM-1	Jensen et al., 2012
Wnt3	Chick wing NMJ, cultured myotubes	AChR clustering	PCP (Rac1, Rho)	Rattner et al., 1997; Weston et al., 2003; Niehrs, 2006; Henriquez et al., 2008
Wnt11r	Zebrafish	AChR clustering	MuSK, Dvl1	Jing et al., 2009
Wnt3a	Hippocampal neurons	Bassoon clustering	Fz1	Varela-Nallar et al., 2012
	Hippocampal neurons, slice	Excitatory transmission	Fz1	Beaumont et al., 2007; Varela-Nallar et al., 2012
	Sensory neurons	Synapsin clustering	Gsk3β	Krylova et al., 2002
	Cultured myotubes	Reduce AChR clustering	Canonical (β-catenin)	Wang et al., 2008
Wnt5a	Hippocampal neurons	Pre-synaptic differentiation	ROR1/2	Paganoni et al., 2010; Varela-Nallar et al., 2012
		Decrease pre-synapse number or no effect	Unclear	Davis et al., 2008; Farias et al., 2009
		Spine growth	Calcium Wnt	Varela-Nallar et al., 2012
		PSD-95 clustering	PCP (JNK)	Farias et al., 2009
	Hippocampal slice	Synaptic transmission	PCP (JNK)	Farias et al., 2009
	Hippocampal neurons	GABAR insertion and clustering	Calcium Wnt (CaMKII)	Cuitino et al., 2010
	Hippocampal slice	Inhibitory transmission	Calcium Wnt (CaMKII)	Varela-Nallar et al., 2010; Cerpa et al., 2011
		Excitatory transmission	Calcium Wnt (CaMKII)	Varela-Nallar et al., 2010; Cerpa et al., 2011
Wnt7a	Cerebellar granule cells	Synapsin clustering	Dvl1, Gsk3β ?	Hall et al., 2000; Ahmad-Annuar et al., 2006
	Cerebellar slice	Pre-synaptic transmission	Dvl1	Ahmad-Annuar et al., 2006
	Hippocampal neurons	Pre-synaptic differentiation	Fz5	Cerpa et al., 2008; Sahores et al., 2010
		Spine growth, PSD-95 clustering	Calcium Wnt (Dvl1, CaMKII)	Ciani et al., 2011
	Hippocampal slice	Pre-synaptic transmission	Calcium Wnt (Dvl1, CaMKII)	Cerpa et al., 2008; Ciani et al., 2011

### Role of wnts in synaptic transmission and plasticity

Just as the fly NMJ provided important insights into how Wnts regulate synapse formation, further studies utilizing this model system have yielded additional roles for Wnt5 and Wnt1/Wg in synaptic transmission and plasticity. Absence of Wnt5, but not Drl, lowers the amplitude of evoked end-plate junctional currents (EJCs) and lowers the frequency of miniature EJCs (mEJCs), indicating defects in pre-synaptic transmission (Liebl et al., 2008). The *wg* mutant also has suppressed activity-dependent synaptic growth (Ataman et al., 2008). In addition, Wg is a potential negative regulator of homeostatic compensation, where it is inhibited by the paired box protein Pax3/7 homolog gooseberry (Marie et al., 2010). However, the downstream mechanisms remain elusive.

A recent study on the *C. elegans* Wnt CWN-2 at the NMJ provides some mechanistic insight (Jensen et al., 2012). In contrast to another Wnt ligand LIN-44 that inhibits pre-synapse formation, CWN-2 promotes synaptic strength by regulating the translocation of an AChR ACR-16/α7 to the synapse (Jensen et al., 2012). Reduction in AChR enrichment and synaptic current is observed both in the absence of CWN-2 in the motor neuron and during the loss of LIN-17/Frizzled, ROR receptor tyrosine kinase CAM-1 or DSH-1/Dvl in muscles. Other Frizzled receptors and Ryk/Drl are not required for the elevation in post-synaptic strength induced by CWN-2. The identities of the effectors downstream of DSH-1/Dvl responsible for AChR translocation remain to be elucidated.

In addition to the synaptogenic functions of Wnt7a, Wnt5a, and Wnt3a, these ligands also increase synaptic transmission in cerebellar and hippocampal slices. In the mossy fiber-granule cell synapses of Wnt7a/Dvl1 double mutant mice, neurotransmitter release is diminished (Ahmad-Annuar et al., 2006). Wnt7a and post-synaptic Dvl1 also increase the frequency of mEPSCs, indicating larger neurotransmitter release in CA3-CA1 synapses in the hippocampus (Cerpa et al., 2008; Ciani et al., 2011). Ciani and colleagues further observed a CaMKII-dependent increase in the amplitude of mEPSCs in hippocampal neurons induced by Wnt7a, suggesting that this ligand acts through the calcium Wnt pathway in dendrites to augment synaptic strength (Ciani et al., 2011). On the other hand, Wnt5a increases both excitatory and inhibitory synaptic transmission and signals through the PCP and calcium Wnt signaling pathways in hippocampal neurons. Wnt5a and JNK, a component of the PCP pathway, regulate glutamatergic synaptic transmission (Farias et al., 2009). In addition, Wnt5a facilitates LTP by augmenting the proportion of GluN2B-containing NMDARs at the synapse, as well as the amplitude of NMDAR currents through the elevation of calcium and the activation of CaMKII (Varela-Nallar et al., 2010; Cerpa et al., 2011). In contrast, through the same calcium Wnt signaling pathway, Wnt5a also increases GABA_A_ receptor recycling and miniature inhibitory post-synaptic currents (mIPSCs) (Cuitino et al., 2010). Wnt3a is likely to have a similar effect as Wnt5a since blocking its activity decreases LTP in hippocampal slices (Chen et al., 2006). Consistent with the previous finding, Wnt3a enlarges neurotransmitter release through pre-synaptic Frizzled-1 in hippocampal neurons (Varela-Nallar et al., 2009) and enhances excitatory transmission in hippocampal slices (Beaumont et al., 2007).

The studies mentioned above suggest that Wnts increase synaptic function *in vivo* and *in vitro* through the PCP pathway, calcium Wnt signaling, and possibly other pathways (Table 1). However, do Wnts affect neural circuit function? In the developing *Xenopus* optic tectum, Lim and colleagues reported that Wnt secreted from tectal cells enhances visual experience-dependent plasticity of receptive fields of cells in the dorsal tectum (Lim et al., 2010). This suggests that regulation of synapse formation and function by Wnt signaling likely leads to downstream effects on circuit function.

## Transforming growth factor-β

TGF-β signaling is critical for multiple biological processes, including proliferation, development, patterning, and regeneration (Kubiczkova et al., 2012). The TGF-β superfamily consists of more than 30 secreted members in humans that are broadly classified into two ligand subfamilies: the TGF-β-activin-Nodal group and the bone morphogenetic proteins (BMPs) group (Shi and Massague, 2003). Different members signal through distinct subtypes of heterotetrameric receptor complexes composed of specific type I and II receptors, leading to phosphorylation of R-Smads and inducing their binding to Smad4. Upon entering the nucleus, the Smad complex interacts with transcription factors to enhance gene expression. Massague provides a detailed description of TGF-β signaling in two recent reviews (Massague, 2012a,b).

Multiple members of the TGF-β superfamily play a role in the developing nervous system and several are regulated by neuronal activity. For instance, developmental expression of TGF-β in the mammalian neocortex is required for axon initiation *in vivo* and *in vitro* (Yi et al., 2010). Depolarization of primary hippocampal neurons with high levels of potassium or glutamate leads to the release of TGF-β (Specht et al., 2003) and the elevated expression of TGF-β2 and TGF-β3 (Lacmann et al., 2007). In addition, both kainate-induced seizures and HFS augment levels of activin β A mRNA in the hippocampus (Andreasson and Worley, 1995; Inokuchi et al., 1996) while sensory deafferentation of the visual cortex reduces activin β A mRNA levels in cortical neurons in specific layers (Andreasson and Worley, 1995). It was also recently reported that lowering activity in the *C. elegans* AVA command interneuron by exposure to pathogenic bacteria enhances release of TGF-β/DBL-1 (Zhang and Zhang, 2012). This precise regulation of TGF-β and activin levels by synaptic input suggests an activity-dependent function for these TGF-β family members in synaptogenesis, synaptic transmission, and plasticity.

### Role of TGF-β in synaptogenesis

**Figure 3:**
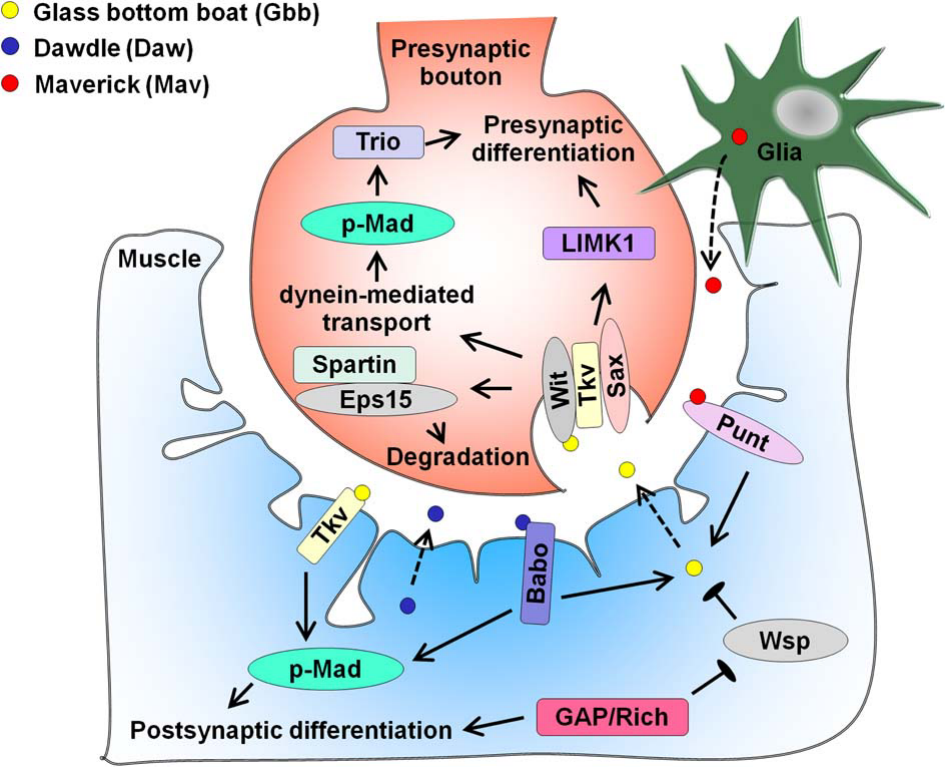
**Bone Morphogenetic Protein (BMP) homolog Glass bottom boat (Gbb), activin ligand Dawdle (Daw), and TGF-β ligand Maverick (Mav) regulate larval NMJ differentiation.** Gbb secreted from the muscle (dotted arrow) signals through pre-synaptic BMP receptors wishful thinking (Wit), thickveins (Tkv), and saxophone (Sax) to enhance synaptogenesis. Gbb binding activates LIMK1 and dynein-mediated retrograde axonal transport of the BMP receptors leads to phosphorylation of the Mad transcription factor, thus driving pre-synaptic differentiation. One of the downstream targets of phosphorylated Mad (p-Mad) is the Rac GEF Trio. Gbb signaling is inhibited by spartin, which binds endocytic adaptor Eps15 and enhances endocytic degradation of Wit. Cysteine-rich transmembrane BMP regulator 1 homolog, the inhibitory Smad Dad, and Highwire are additional negative regulators of Gbb signaling absent from this figure. Gbb also binds Tkv in muscle and regulates gene expression through p-Mad. Apart from Gbb, muscle-derived Daw (dotted arrow) binds Baboon (Babo) to enhance post-synaptic differentiation, while promoting Gbb expression to drive pre-synaptic differentiation. Lastly, glia-derived Mav (dotted arrow) binds Punt and augments Gbb transcription and release. In addition, the Cdc42-selective GAP Rich inhibits the Cdc42 effector Wiskott-Aldrich syndrome protein Wsp, thus stimulating Gbb secretion from the muscle. Rich also promotes post-synaptic development independently of Cdc42.

Studies in the *Drosophila* NMJ have provided key mechanistic insights into how TGF-β family members act as positive regulators of synaptogenesis (Figure 3). Multiple reports from the early 2000s have demonstrated that the BMP homolog Glass Bottom Boat (Gbb) secreted by muscle cells signals through pre-synaptic receptors wishful thinking (Wit), thickveins (Tkv) and saxophone (Sax) to promote NMJ synapse formation (Aberle et al., 2002; Marques et al., 2002; McCabe et al., 2003; Rawson et al., 2003). Gbb binding both activates the LIM-domain kinase LIMK1 to stabilize the synapse (Eaton and Davis, 2005) and phosphorylates the R-Smad transcription factor Mothers against decapentaplegic (Mad) to increase the number of synapses (Rawson et al., 2003). Several downstream targets have been identified, including the Rac guanine nucleotide exchange factor (GEF) Trio (Ball et al., 2010; Kim and Marques, 2010). This signaling requires dynein-mediated retrograde axonal transport of BMP receptors (Smith et al., 2012b). The Tkv receptor and Mad transcription factor are also present in the muscle and may affect post-synaptic development and function (Dudu et al., 2006). To prevent synaptic overgrowth, this pathway is negatively regulated by several factors in the motor neuron, including the cysteine-rich transmembrane BMP regulator 1 homolog that antagonizes BMP signaling (James and Broihier, 2011), the inhibitory Smad Daughters against decapentaplegic (Dad) and the E3 ubiquitin ligase Highwire (McCabe et al., 2004). In addition, the Cdc42 pathway inhibits post-synaptic Gbb secretion (Nahm et al., 2010a,b) while endocytic and endosomal machinery lower surface levels of BMP receptors in neurons (Sweeney and Davis, 2002; Wang et al., 2007; O'Connor-Giles et al., 2008). Gbb secretion is further modulated by HSPG sulfation (Dani et al., 2012). Spartin, which binds to endocytic adaptor Eps15 was recently found to inhibit synaptic growth at the NMJ by promoting endocytic degradation of BMP receptor Wit (Nahm et al., 2013). This leads to elevated levels of fragile X mental retardation protein, a translational repressor of Futsch/microtubule associated protein MAP1B mRNA. Apart from Gbb, the activin ligand Dawdle (Daw) and the TGF-β ligand Maverick (Mav) are also present at the NMJ. Daw acts through the post-synaptic activin type I receptor Baboon (Babo) and Smad2 transcription factor to promote synaptogenesis at the NMJ (Ellis et al., 2010). Daw and Babo further regulate pre-synaptic differentiation by regulating Gbb expression (Ellis et al., 2010). Secreted by glia, Mav regulates synaptic growth by binding muscle activin-type receptor Punt and by increasing Gbb signaling (Fuentes-Medel et al., 2012). Taken together, the BMP homolog Gbb and the activin ligand Daw are potent activators of synapse growth at the NMJ and achieve this by promoting gene expression through the Smad transcription factors. The TGF-β ligand Mav, the activin ligand Daw, and a host of other intracellular components regulate Gbb signaling to ensure strict control of synaptogenesis at the NMJ.

TGF-β family members also enhance synapse formation in mammalian neurons *in vitro*. Treating primary neurons with TGF-β1, activin, or BMP7 augments synapse formation through different effectors. TGF-β1 secreted from astrocytes increases synaptogenesis in cortical neurons by inducing secretion of D-serine, the co-agonist of the NMDAR (Diniz et al., 2012). Activin promotes synaptic development and alters spine morphology in hippocampal neurons by modulating actin dynamics. This process is independent of protein and RNA synthesis (Shoji-Kasai et al., 2007). BMP7 accelerates hippocampal dendrite development and elevates the rate of synaptogenesis, but the underlying mechanism remains unclear (Withers et al., 2000). Though these findings indicate a synaptogenic role for TGF-β1, activin, and BMP7, it is uncertain if these TGF-β family members function likewise *in vivo* in the vertebrate CNS. Recently, Xiao and colleagues examined the auditory system of conditional BMPR1a and BMPR1b double knockout mice and observed smaller synapses with fewer docked synaptic vesicles, as well as multiple inputs, at the calyx of Held (Xiao et al., 2013). Hence, BMP signaling regulates synapse size and elimination *in vivo* at the calyx of Held.

While other TGF-β family members have primary roles in driving synaptogenesis, *Drosophila* activin (dactivin) and myoglianin, a *Drosophila* TGF-β2 ligand, are involved in synaptic patterning in the visual system and NMJ, respectively (Ting et al., 2007; Awasaki et al., 2011; Yu et al., 2013). In the *Drosophila* visual system, mutations in Babo and the Smad2-interacting nuclear import protein importin-α3 lead to overlap of R7 photoreceptor axon terminals with those in neighboring columns (Ting et al., 2007). Similar defects in tiling occur in the absence of dactivin or Smad2. Hence, activin regulates activity of Smad2 to ensure formation of appropriate pre-synaptic contacts. In the larval NMJ, TGF-β2/myoglianin secreted from muscle acts through Babo to prevent formation of ectopic synapses and this process is regulated by the immunoglobulin superfamily transmembrane protein Plum, as well as the ecdysone receptor-B1 (Yu et al., 2013). However, the downstream signaling mechanism has not been characterized.

### Role of TGF-β in synaptic transmission and plasticity

In addition to driving synaptogenesis, members of the TGF-β family are implicated in promoting excitatory synaptic transmission. Work on long-term synaptic facilitation in the marine mollusk *Aplysia californica* provided the earliest evidence of the ability of TGF-β1 to sculpt synaptic transmission (Zhang et al., 1997). This was followed by the finding that TGF-β1 induces long-term increases in neuronal excitability by activating mitogen-activated protein kinase (MAPK), a well-established regulator of LTP in *Aplysia* (Chin et al., 2006). TGF-β1 also acutely activates MAPK, altering distribution of the pre-synaptic protein synapsin and reducing synaptic depression in the *Aplysia* sensorimotor synapse (Chin et al., 2002). Treatment of cultured hippocampal neurons with TGF-β2 also led to an analogous effect—decreased short-term synaptic depression of evoked post-synaptic currents (Fukushima et al., 2007). This observation is associated with heightened phosphorylation of cAMP response element-binding protein (CREB). Consistent with a role for TGF-β2 in promoting synaptic transmission, TGF-β2 knockout mice have impaired transmission in GABAergic/glycinergic and glutamatergic synapses in the brainstem where both frequency of mEPSCs and total charge transfer are suppressed (Heupel et al., 2008). This effect on pre-synaptic transmission by TGF-β1 and TGF-β2 is reminiscent of diminished neurotransmitter release in the fly NMJ when BMP signaling is disrupted, and this process is likely partially mediated through the lymphocyte antigen 6 (Ly6) neurotoxin-like molecule target of Wit (Aberle et al., 2002; Marques et al., 2002; Baines, 2004; McCabe et al., 2004; Nahm et al., 2010b; Kim and Marques, 2012). Conversely, chordin null mice that have elevated BMP signaling exhibit augmented pre-synaptic neurotransmitter release, as reflected from enhanced paired pulse facilitation (PPF) and LTP (Sun et al., 2007). This observation is unlikely due to transduction through Smad4 since Smad4 knockout mice have stronger, instead of weaker, PPF in excitatory synaptic transmission in the hippocampus (Sun et al., 2010). In addition, at the calyx of Held synapse, knocking out both BMPR1a and BMPR1b reduced the amplitude of EPSCs and lengthened decay times, indicating that a loss in BMP signaling reduces synaptic transmission (Xiao et al., 2013).

Besides TGF-β1, TGF-β2, and BMP, activin also enhances excitatory synaptic transmission. In cultured hippocampal neurons, activin phosphorylates NMDARs, possibly inducing LTP (Kurisaki et al., 2008). This signaling occurs through Src family tyrosine kinases, PDZ proteins, and activin receptor interacting protein 1. Coherent with this finding, transgenic mice with impaired activin function have reduced NMDA currents and LTP in glutamatergic synapses in the hippocampus (Muller et al., 2006). Similarly, inhibiting activin by overexpressing follistatin in mouse forebrain neurons also impairs hippocampal late-LTP and long-term memory formation during contextual fear conditioning (Ageta et al., 2010). What are the downstream mediators that induce LTP in the presence of BMP and activin? As Smad4-deficient mice do not exhibit defects in LTP or spatial memory, it is possible that BMP and activin regulate hippocampal LTP through non-canonical signaling pathways that might include MAPK (Zhou et al., 2003; Sun et al., 2010).

Activin also suppresses inhibitory synaptic transmission, but this may occur through the canonical Smad-dependent pathway (Krieglstein et al., 2011). Impairing activin function by expressing a dominant-negative mutant of activin receptor in forebrain neurons enhanced spontaneous GABA release and GABA_B_ receptor function in hippocampal neurons and suppressed anxiety-like behavior in mice (Zheng et al., 2009). Since Smad4 knockout mice have larger paired-pulse depression of GABA_A_ currents in the hippocampus (Sun et al., 2010), it is possible that activin regulates GABAergic synapses through Smad4. Lastly, activin indirectly affects the excitatory-inhibitory balance by decreasing the number of GABAergic interneurons while increasing that of dentate gyrus granule cells (Sekiguchi et al., 2009). Taken together, the TGF-β superfamily enhances excitatory synaptic transmission, while suppressing inhibitory synaptic transmission. The downstream effectors differ for the different members and likely involve both Smad-dependent and Smad-independent pathways. Several other reviews cover further details on the effect of TGF-β on synapses and behavior (Krieglstein et al., 2011; Salinas, 2012).

## Tumor necrosis factor-α

TNF-α is a type II transmembrane 26 kDa precursor molecule which is proteolytically cleaved by the metalloprotease TNF-α converting enzyme, a disintegrin and metallopeptidase domain 17 (ADAM17) to generate a soluble 17 kDa homotrimeric pro-inflammatory cytokine (Horiuchi et al., 2010). Both membrane-bound and soluble forms of TNF-α contribute to a broad range of physiological and pathological activities, including cell proliferation, differentiation, apoptosis, and inflammatory responses in various cells (Wang et al., 2005; Chapard et al., 2012).

TNF-α is secreted by a variety of cells such as macrophages, monocytes, neutrophils, T cells, natural killer cells, adipocytes, and fibroblasts (Fahey et al., 1995; Jovinge et al., 1996; Cawthorn and Sethi, 2008; Ambler et al., 2012; Brotas et al., 2012; Zakka et al., 2012), and its signaling is transduced through TNF receptor 1 (TNFR1) and TNF receptor 2 (TNFR2). Soluble TNF-α binds preferentially to TNFR1, which is expressed in neurons (Brambilla et al., 2011) whereas transmembrane TNF-α binds to TNFR2, which is mainly expressed in immune cells such as those of the myeloid lineage, lymphocytes, and macrophages (McCoy and Tansey, 2008).

TNFRs regulate both cell death and survival depending on the cellular environment and context. Activation of TNFR1 recruits the intracellular death domain (DD)-containing adaptor TNFR-associated DD protein (TRADD), which can also recruit the receptor-interacting protein kinase 1 (RIPK1) and TNFR-associated factor-2 (TRAF2). This complex leads to the activation of the transcription factor AP-1 through MAPK and JNK pathways that prevent the triggering of cell death processes. In contrast, TRADD can also promote the recruitment of the Fas-associated DD protein (FADD), which is associated with a caspase-dependent or caspase-independent cell death signaling process known as apoptosis or necrosis, respectively (Chu, 2013). These cell death processes require the internalization of the TNFR (Schneider-Brachert et al., 2004).

Numerous studies have recently shown that TNF-α is involved in inflammatory events in the CNS and have opposing effects depending on their levels in the brain (Hoffmann et al., 2009; Mc Guire et al., 2011; Smith et al., 2012a). TNF-α is secreted by non-neural cells in the brain, including activated astrocytes and microglial cells (Santello and Volterra, 2012) in response to pathological brain conditions and diseases, which can play a protective role in neurons. Under physiological conditions, TNF-α controls the inflammatory response, hence defending against infection. However, excessive amounts of TNF-α are indicative of acute and chronic neuroinflammation. Not surprisingly, TNF-α is involved in several neurodegenerative disorders associated with neuroinflammation and neuronal cell death such as Alzheimer's disease (AD), Parkinson's disease, and HIV-associated dementia (Brabers and Nottet, 2006; Frankola et al., 2011). Consistent with these reports, chronic expression of neuronal TNF-α enhances neuronal cell death in an AD mouse model (Janelsins et al., 2008).

Since the effect of TNF-α signaling is largely dependent on its concentration, multiple factors including neuronal activity, excitotoxicity, and neuroinflammation are involved in TNF-α regulation. Elevating neuronal activity by whisker stimulation elevates TNF-α expression in the somatosensory cortex, as measured by immunostaining (Churchill et al., 2008). Excitotoxicity induced by chronic treatment of NMDA also enhances the expression of TNF-α and other neuroinflammatory markers (Chang et al., 2008). Lastly, treatment with lipopolysaccharide (LPS) augments the expression of TNF-α (Ikeda et al., 2007; Dholakiya and Benzeroual, 2011; Welser-Alves and Milner, 2013). TNF-α released from microglia and astrocytes up-regulates gene transcription for arachidonic acid (AA) cascade enzymes via the nuclear factor kappa B (NF-κ B) pathway, which has been shown to damage neurons by activating pro-apoptotic factors and caspase-3 (Rao et al., 2012).

**Figure 4:**
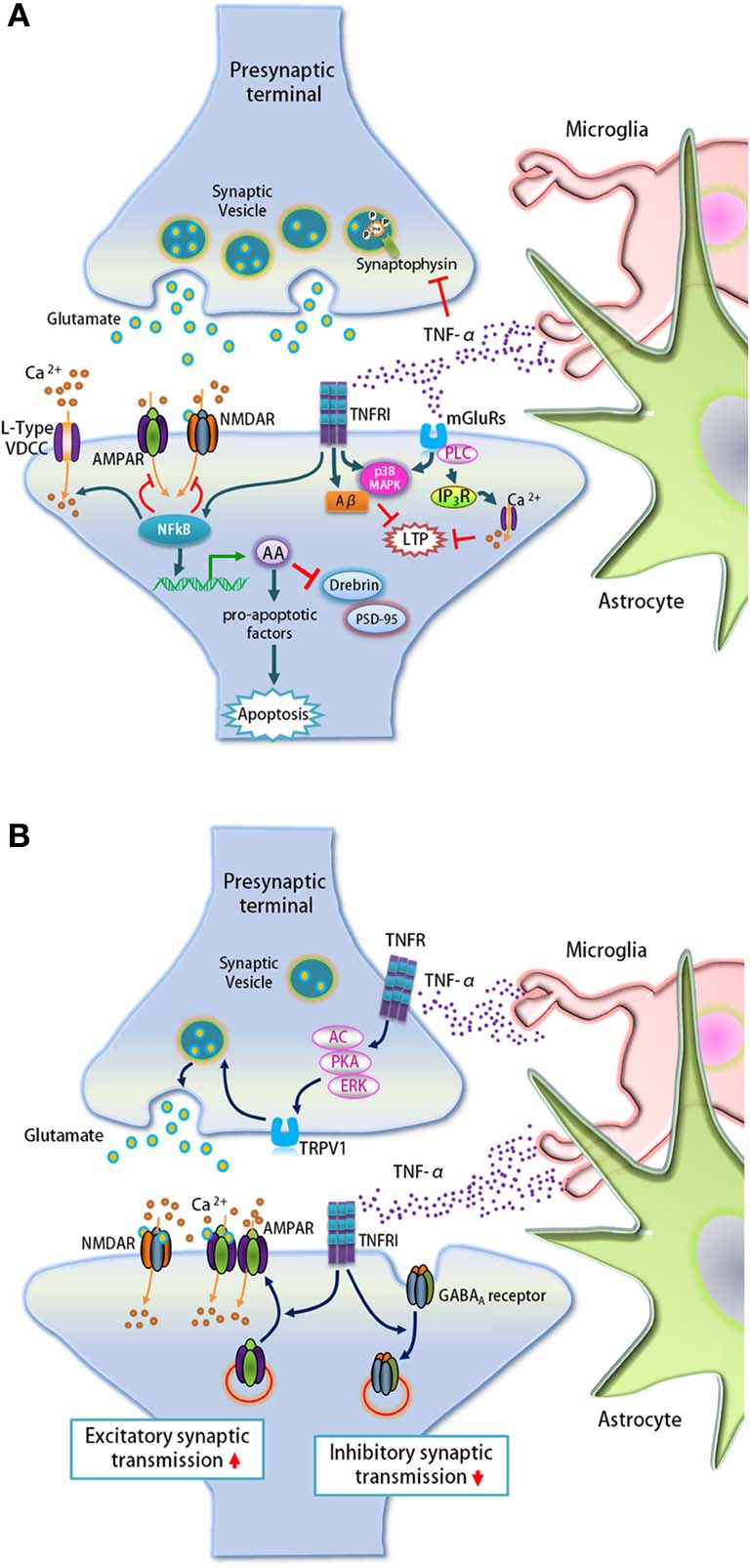
**TNF-α regulates synaptic loss, synaptic transmission, and plasticity. (A)** TNF-α secreted by microglia and astrocytes up-regulates arachidonic acid (AA) via the NF-κ B pathway that leads to synaptic loss by activating pro-apoptotic factors. The elevated AA signaling also down-regulates the expression of post-synaptic proteins drebrin and PSD-95, and pre-synaptic protein synaptophysin. In addition, TNF-α regulates intracellular calcium levels through the NF-κ B pathway by regulating calcium entry through VDCCs, while suppressing glutamate receptor agonist-induced currents in hippocampal neurons. Lastly, TNF-α blocks the early phase of LTP through the activation of TNFR1 and mGluRs and this process is dependent on p38 MAPK, IP_3_ receptor-mediated calcium release via PLC and Aβ. **(B)** Glia-derived TNF-α signals through pre- and post-synaptic TNFR1 to regulate pre-synaptic neurotransmitter release and the surface expression of AMPARs and GABA_A_ receptors, respectively. In spinal cord outer lamina II neurons, TNF-α increases spontaneous EPSC frequency via TRPV1-mediated glutamate release that is mediated by AC, PKA, and ERK pathways from pre-synaptic terminals. At the post-synapse, TNF-α promotes GluA2-lacking AMPAR trafficking to the surface and induces GABA_A_ receptor endocytosis.

During neuroinflammation, an elevation in TNF-α levels and AA signaling alters synaptic protein expression and leads to the loss of synapses (Figure 4A). LPS-induced neuroinflammation lowers the protein levels of several key molecules including the pre-synaptic vesicle protein synaptophysin, the neuron-specific post-synaptic F-actin-binding protein drebin, and PSD-95 (Kellom et al., 2012; Rao et al., 2012). Reductions in these pre- and post-synaptic proteins suggest that high TNF-α levels induced by neuroinflammation may enhance synaptic loss. Furthermore, synaptic loss induced by LPS is abolished in neurons cultured with microglia that produce less TNF-α, indicating that TNF-α mediates LPS-induced synapse loss (Xing et al., 2011; Kellom et al., 2012).

### Role of TNF-α in synaptic transmission and plasticity

TNF-α has been reported to play important roles in neuronal functions such as microglia activation, synaptic transmission, and synaptic plasticity (Stellwagen et al., 2005; Watters and O'Connor, 2011). Activated astrocytes and microglia increase the expression and secretion of TNF-α (Santello and Volterra, 2012), and also promote glutamatergic excitatory synaptic transmission and plasticity (Stellwagen and Malenka, 2006; Kawasaki et al., 2008; Wheeler et al., 2009; Steinmetz and Turrigiano, 2010; Park et al., 2011a; Zhang and Dougherty, 2011; Zhang et al., 2011; O'Connor, 2013).

TNF-α was shown to regulate calcium currents at the post-synapse through the NF-κ B pathway (Furukawa and Mattson, 1998), as well as block LTP in the hippocampus (Butler et al., 2004; Pickering et al., 2005) (Figure 4A). In cultured hippocampal neurons, long-term but not short-term treatment with TNF-α augments calcium currents through post-synaptic L-type voltage-dependent calcium channels (VDCCs), and decreases glutamate receptor agonist-induced currents. In addition, TNF-α blocks the early phase of LTP but not the late phase through the activation of TNFR1 and metabotropic glutamate receptors (mGluRs) in a p38 MAPK–dependent manner (Butler et al., 2004; Pickering et al., 2005). TNF-α activation of mGluRs leads to inositol-1,4,5-trisphosphate (IP_3_) receptor-mediated calcium release via phospholipase C (PLC), which elevates intracellular calcium concentration to impair LTP (Pickering et al., 2005). Group I/II mGluR antagonist MCPG and the selective mGluR5 antagonist MPEP significantly attenuate the inhibition of LTP by TNF-α (Cumiskey et al., 2007). The inhibition of LTP by TNF-α was significantly reversed by ryanodine, which blocks the release of intracellular calcium from ryanodine-sensitive stores. This implicates the involvement of ryanodine-sensitive intracellular calcium stores in TNF-α-mediated inhibition of LTP (Cumiskey et al., 2007).

Apart from impairing LTP by regulating calcium stores, TNF-α is also involved in LTP inhibition mediated by amyloid-β (Aβ), a major component of plaques in AD brains (Figure 4A). Many studies have shown that hippocampal LTP is blocked by Aβ (Cullen et al., 1997; Lambert et al., 1998; Itoh et al., 1999; Chen et al., 2000; Stephan et al., 2001; Vitolo et al., 2002; Walsh et al., 2002; Raymond et al., 2003; Wang et al., 2005; Kotilinek et al., 2008; Jo et al., 2011; Li et al., 2011; Kimura et al., 2012; Olsen and Sheng, 2012; Li et al., 2013b). In addition, expression of TNF-α and its receptor TNFR1 is up-regulated in the brain and plasma of AD patients (Tarkowski, 2002; Li et al., 2004a). Using mutant mice null for TNFR1 and TNF-α, as well as inhibitors, Wang and colleagues reported that TNF-α and TNFR1 are required for Aβ-mediated LTP inhibition. This TNF-α-mediated inhibition of LTP is dependent on the activation of p38 MAPK and mGluR5 (Wang et al., 2005).

While the earlier studies focused on how pathological levels of TNF-α impair LTP in the hippocampus, the role of TNF-α in the spinal cord has also been explored. In several models of neuropathic pain, expression of TNF-α and TNFR1 is up-regulated in the spinal dorsal horn (Ikeda et al., 2007; Wei et al., 2007), and elevated levels of TNF-α induce spinal LTP in a JNK-, p38 MAPK-, and NF-κ B-dependent fashion (Liu et al., 2007). Inhibition of TNF-α signaling abolishes LTP (Zhong et al., 2010), and intriguingly, inhibition of Src-family kinases leads to HFS-induced LTD, instead of LTP, and this inhibitory effect on spinal LTP is reversed by TNF-α addition (Zhong et al., 2010).

In addition to its effect on synaptic proteins, calcium levels, and LTP, TNF-α also enhances excitatory synaptic transmission and suppresses inhibitory synaptic transmission by regulating the surface expression of post-synaptic receptors (Figure 4B). Immunocytochemistry and electrophysiology revealed that treatment with TNF-α or astrocyte-derived conditioned media containing TNF-α elevates the surface levels of α-amino-3-hydroxy-5-methyl-4-isoxazolepropionic acid receptors (AMPARs) as well as the frequency of mEPSCs in cultured hippocampal neurons (Beattie et al., 2002). Glial TNF-α signaling through TNFR1 was shown to be involved in this AMPAR-mediated control of synaptic strength (Beattie et al., 2002). Furthermore, genetic approaches have shown that deletion of TNFR1 but not TNFR2 lowers the surface expression and synaptic localization of AMPARs, suggesting a critical role of TNFR1 signaling in AMPAR-mediated synaptic functions (He et al., 2012). Intriguingly, the effect of TNF-α on surface AMPARs preferentially affects GluA2-lacking AMPARs (Stellwagen et al., 2005). In contrast to its effect on AMPARs, TNF-α induces GABA_A_ receptor endocytosis, diminishing surface expression of GABA_A_ receptors and inhibitory synaptic strength (Stellwagen et al., 2005). Taken together, TNF-α affects both excitatory and inhibitory synaptic transmission, suggesting an important role of TNF-α in the homeostasis of neural circuits.

In addition, TNF-α also enhances synaptic transmission in the spinal cord. In spinal cord outer lamina II neurons, TNF-α increases spontaneous EPSC frequency but not amplitude via pre-synaptic transient receptor potential subtype V1 (TRPV1)-mediated glutamate release that is dependent on adenylyl cyclase (AC), PKA, and the extracellular signal-regulated kinase (ERK) in pre-synaptic terminals (Park et al., 2011a). However, this spinal cord LTP induction is abolished in Tnfr1^−/−^ mice, Tnfr2^−/−^ mice, and Trpv1^−/−^ mice. This observation indicates the importance of TNFR and TRPV1 in spinal cord LTP (Park et al., 2011a).

## Perspectives

Growth factors like netrin, Wnt, TGF-β, and TNF-α were first identified for their roles in axon guidance, embryonic development, cell proliferation, and inflammation, respectively. Over the past decade, they have gained prominence as regulators of the synapse. Similar to other patterning molecules such as sonic hedgehog (Salie et al., 2005), these growth factors play multiple roles during development. By utilizing effectors that multi-task, the nervous system can carry out multiple functions more efficiently. For a single factor that has to fulfill various roles, diverse regulatory and signaling pathways that are spatiotemporally restricted must be put in place for it to achieve distinct functional outcomes.

Synapse formation largely involves transport, recruitment, and assembly of molecular machinery, cytoskeletal remodeling, and eventual stabilization of the synapse. As described earlier, netrin, Wnt, TGF-β, and TNF-α largely promote synaptogenesis and/or synaptic transmission but several including netrin/UNC-6, Wnt5a, Wnt4, Wnt3a, Wnt/LIN-44, TGF-β2, and activin also act as negative regulators. It is intriguing to note how some of these factors have opposing effects on synaptogenesis. In the case of netrin, the use of distinct receptors—DCC/UNC-40 or UNC-5 determine its effect on the synapse (Colon-Ramos et al., 2007; Poon et al., 2008). For the Wnt family, only a few members have inhibitory effects on synaptogenesis and distinct pathways are utilized for this purpose (Table 1). Lastly, only two members of the TGF-β family negatively regulate synaptogenesis: dactivin and myoglianin in *Drosophila* (Ting et al., 2007; Yu et al., 2013). As a pro-synaptogenic role for both these ligands has yet to be identified, they may activate pathways to specifically inhibit ectopic synapse formation. In addition, a putative mechanism coordinating synapse formation and elimination within a single neuron is discussed in a recent paper by Park and colleagues (Park et al., 2011b).

Apart from the opposing effects of several growth factors on synaptogenesis, TNF-α also appears to have conflicting effects on LTP and surface levels of AMPARs. Many studies have reported that an increased level of TNF-α impairs LTP in the hippocampus (Butler et al., 2004; Cumiskey et al., 2007; Liu et al., 2007, 2012) and also elevates the surface level of GluA2-lacking Ca^2+^-permeable AMPARs in cultured hippocampal neurons (Beattie et al., 2002; Stellwagen et al., 2005). It is important, however, to note that TNF-α increases the insertion of Ca^2+^-permeable AMPARs to both synaptic and extrasynaptic sites (Ferguson et al., 2008; Leonoudakis et al., 2008). In addition, Ca^2+^-permeable AMPARs are incorporated into the surface during LTP (Plant et al., 2006). Thus, one plausible mechanism is that TNF-α elevates the surface level of AMPARs at both synaptic and extrasynaptic sites, leading to excessive calcium influx through synaptic and extrasynaptic AMPARs, hence impairing LTP.

Considering how the synapse is key to proper communication between neurons, one would expect a complex interplay of multiple molecular mechanisms to ensure tight regulation of synaptic function. Studies in the *C. elegans* AIY interneuron and the *Drosophila* NMJ have provided strong mechanistic insights into how netrin, Wnt, and TGF-β regulate synaptic function. However, it remains unclear if these growth factors utilize similar pathways in mammals. Identification of the target genes that lie downstream of the different signaling pathways will elucidate how the diverse growth factors differentially regulate synaptic function.

Given that synaptic function is compromised in a majority of neurological diseases, further understanding of the signaling pathways of netrin, Wnt, TGF-β, and TNF-α may contribute to novel therapeutic approaches for these debilitating disorders.

### Conflict of interest statement

The authors declare that the research was conducted in the absence of any commercial or financial relationships that could be construed as a potential conflict of interest.

## Abbreviations

AA: arachidonic acid
ABI-1: Abl-interacting protein-1
Abl: Abelson tyrosine-protein kinase 1
AC: adenyl cyclase
AChR: acetylcholine receptor
ACR-: acetylcholine receptor
AD: Alzheimer's disease
ADAM17: a disintegrin and metallopeptidase domain 17
AMPAR: α-amino-3-hydroxy-5-methyl-4-isoxazolepropionic acid receptor
APC: adenomatous polyposis coli
Aβ: amyloid-β
Babo: baboon
BMP: bone morphogenetic protein
BMPR: BMP receptor
CAM-: CAN cell migration defective
CaMKII: calcium/calmodulin-dependent protein kinase II
Cdc42: cell division cycle 42
CDK-5: cyclin-dependent kinase 5
CED-: cell death abnormality
CNS: central nervous system
CREB: cAMP response element-binding protein
CWN-: *C. elegans* WNT family
CYY-: cyclin Y
dactivin: *Drosophila* activin
Dad: daughters against decapentaplegic
Daw: dawdle
DBL-: decapentaplegic/BMP-like
DCC: deleted in colorectal cancer
DD: death domain
DFz2: *Drosophila* Frizzled-2
DGRIP: *Drosophila* glutamate receptor interacting protein
DOCK180: dedicator of cytokinesis 1
Drl: derailed
DSCAM: down syndrome cell adhesion molecule
DSH-: dishevelled related
Dvl: dishevelled
EJC: end-plate junctional current
Eps15: epidermal growth factor receptor pathway substrate 15
ERK: extracellular signal-regulated kinase
Evi/Wls/Srt: Evenness Interrupted/Wntless/Sprinter
FADD: fas-associated DD
FNI: frizzled nuclear import
GABA_A,_: γ-aminobutyric acid A
GAP: guanosine triphosphatase-activating protein
Gbb: glass bottom boat
GEF: guanine nucleotide exchange factor
GluN2B: an NMDAR subunit
GSK3β: glycogen synthase kinase 3β
HIV: human immunodeficiency virus
HFS: high frequency stimulation
HSPG: heparan sulfate proteoglycan
IP_3_: inositol-1,4,5-trisphosphate
JNK: c-Jun-amino-terminal kinase
LIMK1: LIM domain kinase 1
LIN-: abnormal cell lineage
LPS: lipopolysaccharide
LTD: long-term depression
LTP: long-term potentiation
Ly6: lymphocyte antigen 6
Mad: mothers against decapentaplegic
MAP: microtubule associated protein
MAPK: mitogen-activated protein kinase
Mav: maverick
MCPG: α-methyl-4-carboxyphenylglycine
mEJC: miniature end-pate junctional current
mEPSC: miniature excitatory postsynaptic current
mGluR: metabotropic glutamate receptor
MIG-: abnormal cell migration
mIPSC: miniature inhibitory postsynaptic current
MPEP: 2-methyl-6-(phenylethynyl)pyridine
MuSK: muscle-specific kinase
NF-κ B: nuclear factor kappa B
NFAT: nuclear factor of activated T-cells
NGL: netrin G ligand
NMDAR: N-methyl-D-aspartate-type glutamate receptor
NMJ: neuromuscular junction
PCP: planar cell polarity
PCT-1: Pctaire kinase 1
PKA: protein kinase A
PKC: protein kinase C
PLC: phospholipase C
PPF: paired pulse facilitation
PSD-95: postsynaptic density protein 95
Rac1: ras-related C3 botulinum toxin substrate 1
RIPK1: receptor-interacting protein kinase 1
ROR: receptor tyrosine kinase-like orphan receptor
Ryk: receptor-like tyrosine kinase
Sax: Saxophone
Smad: Mad homolog
Src: Rous sarcoma oncogene
TGF-β: transforming growth factor-β
Tkv: Thickveins
TNF-α: tumor necrosis factor-α
TNFR: TNF receptor
TRADD: TNFR-associated DD protein
TRAF2: TNFR-associated factor-2
TRPV1: transient receptor potential subtype V1
UNC-: Uncoordinated
VDCC: voltage-dependent calcium channel
VTA: ventral tegmental area
Wg: Wingless
Wit: wishful thinking.
